# Cellular and Molecular Mechanism of Cardiac Regeneration: A Comparison of Newts, Zebrafish, and Mammals

**DOI:** 10.3390/biom10091204

**Published:** 2020-08-19

**Authors:** Lousanne de Wit, Juntao Fang, Klaus Neef, Junjie Xiao, Pieter A. Doevendans, Raymond M. Schiffelers, Zhiyong Lei, Joost P.G. Sluijter

**Affiliations:** 1 Department of Cardiology, Experimental Cardiology Laboratory, UMC Utrecht, 3584CX Utrecht, The Netherlands; l.h.c.dewit@students.uu.nl (L.d.W.); J.Fang-2@umcutrecht.nl (J.F.); K.neef-2@umcutrecht.nl (K.N.); P.Doevendans@umcutrecht.nl (P.A.D.); 2 UMC Utrecht RM Center, Circulatory Health Laboratory, 3584CT Utrecht, The Netherlands; 3 Institute of Cardiovascular Sciences, Shanghai University, Shanghai 200444, China; junjiexiao@live.cn; 4 Utrecht University, 3584CS Utrecht, The Netherlands; 5 Netherlands Heart Institute (NHI), Central Military Hospital (CMH), 3511EP Utrecht, The Netherlands; 6 Division LAB, CDL Research, UMC Utrecht, 3584CX Utrecht, The Netherlands; R.Schiffelers@umcutrecht.nl

**Keywords:** cardiac regeneration, cardiomyocyte dedifferentiation, proliferation, cardiomyocyte polyploidy

## Abstract

Cardiovascular disease is the leading cause of death worldwide. Current palliative treatments can slow the progression of heart failure, but ultimately, the only curative treatment for end-stage heart failure is heart transplantation, which is only available for a minority of patients due to lack of donors’ hearts. Explorative research has shown the replacement of the damaged and lost myocardium by inducing cardiac regeneration from preexisting myocardial cells. Lower vertebrates, such as the newt and zebrafish, can regenerate lost myocardium through cardiomyocyte proliferation. The preexisting adult cardiomyocytes replace the lost cells through subsequent dedifferentiation, proliferation, migration, and re-differentiation. Similarly, neonatal mice show complete cardiac regeneration post-injury; however, this regenerative capacity is remarkably diminished one week after birth. In contrast, the adult mammalian heart presents a fibrotic rather than a regenerative response and only shows signs of partial pathological cardiomyocyte dedifferentiation after injury. In this review, we explore the cellular and molecular responses to myocardial insults in different adult species to give insights for future interventional directions by which one can promote or activate cardiac regeneration in mammals.

## 1. Introduction

Cardiovascular disease is the leading cause of death worldwide [1], of which 13.7% of all cardiovascular deaths in 2015 suffered from acute myocardial infarction (AMI). AMI can lead to loss of more than one billion cardiomyocytes [2], and within days to a few weeks, a collagen-rich scar is formed. This scar, in combination with the loss of cardiomyocytes, results in a decline of cardiac contractility, driving further cardiac remodeling, reducing ventricular ejection fraction, and can even lead to sudden cardiac death [2]. Most of the treatments, such as interventional therapies (e.g., coronary angioplasty, stent placement), pharmacological agents (e.g. β-adrenergic and angiotensin receptor blockers) and invasive surgical therapy (e.g., left ventricular assist device) are palliative. The only curative therapy for end-stage heart failure is heart transplantation. However, it is an invasive procedure and the demand greatly exceeds the limited number of donor hearts available [3].

Because of this, many attempts to prevent massive cell loss during ischemic events, and thereby retaining myocardial function, have been initiated, including cell death inhibitors [4], modulation of the immune system [5], and reperfusion injury strategies [6]. Since many attempts did not obtain clinically relevant improvements, other strategies have been extensively tested to repopulate the myocardium, including tissue engineering approaches [7], the use of cellular paracrine signals [8] and cell transplantation studies [9]. The latter has the ultimate goal to recruit or transplant stem or progenitor cell-derived cardiomyocytes to the injury site, where they should integrate or secrete factors that stimulate tissue healing and regeneration. Unfortunately, limited successes in prolonged, improved myocardial contractility have been reported. From groundbreaking observations by Bergmann et al. [10], we know that the adult heart has a limited myocardial turnover that can replace cardiomyocytes throughout a lifetime. Therefore, researchers started to look into endogenous approaches for the regeneration of adult mammalian hearts.

Some vertebrate species from the amphibian and bony fish classes, such as newts and zebrafish, are capable of cardiomyocyte proliferation [11,12,13] and regeneration of their hearts upon injury with transient scarring [2,14,15], while adult mammals develop dysfunctional scar tissue post-myocardial injury [2]. Neonatal mammals, such as mice and humans [16], display some capacity to regenerate their heart after injury [17,18,19,20], but these regenerative processes differ from mechanisms in adults. Readers interested in neonatal regeneration are referred to recent reviews on this topic [21].

**Figure 1:**
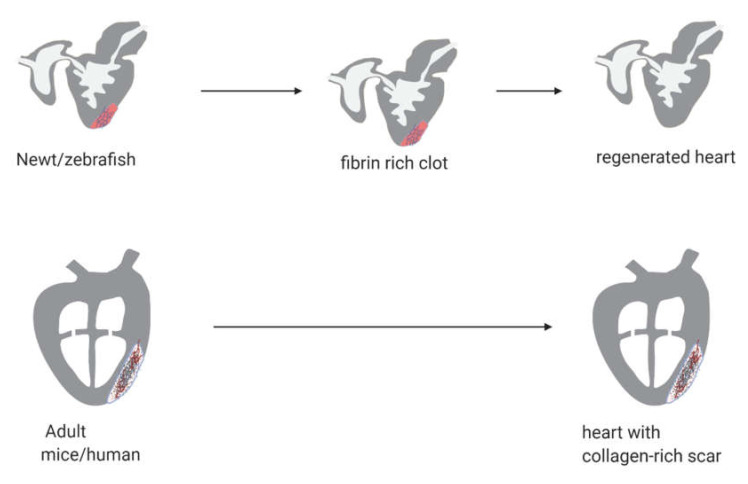
Cardiac regeneration in newts, zebrafish and mammals (mice and human). Upon partial resection of the heart, newts and zebrafish show the development of a blood clot to stop the bleeding. Their cardiomyocytes start to dedifferentiate and immune cells, especially macrophages, infiltrate the injured area. The blood clot will gradually become a fibrin clot. Simultaneously, the dedifferentiated cardiomyocytes will start proliferating to replace the fibrin clot. After a few days, the resected tissue will be fully replaced by functional heart tissue. Adult mammals, like mouse and human, on the other hand, cannot regenerate their hearts after myocardial injury. They show high levels of cardiomyocyte cell death, necrosis, and apoptosis, and infiltration of neutrophils into the injured area. Some cardiomyocytes will show signs of partial, pathological dedifferentiation, but adult mammalian cardiomyocytes cannot proliferate in response to injury. Ultimately, a collagen-rich scar will be formed. The picture is created with BioRender.com.

**Figure 2:**
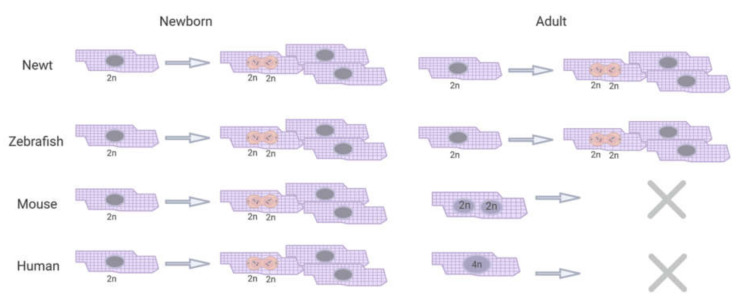
Total DNA content per cardiomyocyte in relation to their proliferative capacity. Newt and zebrafish cardiomyocytes stay mononucleated, diploid, and proliferative their whole life. Newborn mouse and human cardiomyocytes have similar characteristics as newt and zebrafish, but the mouse cardiomyocytes become binucleated and human cardiomyocytes become tetraploid when they become adults. Simultaneously, these cardiomyocytes lose the ability to proliferate.

Newt, zebrafish, and mammalian hearts have diverse anatomical and physiological characters [14,18,22]. Newts and zebrafish, so-called poikilothermic vertebrates, have a trabecular ventricle with a spongy lumen (Figure 1). This helps cardiomyocytes obtain oxygen directly from the bloodstream. A newt ventricle can, therefore, be avascular and its heart consists of two atria and a single ventricle [14]. Zebrafish hearts have only one atrium and ventricle, but in contrast to newts, their ventricle is vascularized [22]. Moreover, the myocardial wall consists of an epicardium, myocardium and endocardium [23]. Homeothermic mammals have a more compact myocardium and a more distinct central lumen, making coronary vascularization essential for oxygen supply to the cardiomyocytes [24]. Furthermore, cardiomyocytes from lower vertebrates and mammals differ in their ability to enter the cell cycle as shown in Figure 2. Ninty-nine percent of the cardiomyocytes in adult zebrafish are mononucleated and can re-enter the cell cycle [25]. Cardiomyocytes in newborn humans and mice are also mainly diploid [10,26,27]; their total DNA content per cardiomyocyte changes shortly after birth. Within the first week after birth, 95% of mouse cardiomyocytes become binucleated [27,28] and human cardiomyocytes become tetraploid during the first month after birth [10,26]. Mammalian adult hearts have been considered postmitotic organs for a long time. Numerous mitotic cells were found in the mammalian hearts before birth, while this proportion quickly dropped after birth, as is indicated by terminal differentiation and only minimal renewal rate of cardiomyocytes could be observed in many studies [28]. Although DNA replication in cardiomyocytes has been shown during different cardiac developmental stages and post-injury, actual cytokinesis in cardiomyocytes remains largely unclear [29,30]. Interestingly, a lineage tracing study performed in adult zebrafish demonstrated that the majority of newly formed cardiomyocytes in response to injury merely come from preexisting cardiomyocytes [11,15]. These observations brought interest in generating new cardiomyocytes from preexisting adult human cardiomyocytes. Therefore, re-activation of similar regenerative pathways in the adult mammalian heart offers potential new opportunities for curative therapeutic approaches in cardiac disease, restoring lost functional myocardium. Within this review, we provide an overview of the regenerative responses in newts and zebrafish, along with the mammalian response, describe the key factors that are involved and propose a working model for adult cardiac regeneration.

## 2. Cardiac Regeneration in Experimental Animal Models

When comparing vertebrate species, a loss of cardiac regenerative potential is observed in mammals [31]. This observation is not limited to the myocardium but is present in various tissues and organs. During a cardiac regenerative response, signaling from the extracellular matrix (ECM) exerts a vital role in cellular migration, proliferation, and differentiation of cardiomyocytes [32]. Compared to adult mammals, newt and zebrafish undergo a large scale cardiac remodeling process, including diminished scar formation but with substantial ECM deposition [18,33,34]. In newts and zebrafish, the genes of ECM components are one of the most responsively expressed cardiac gene families after a myocardial injury. The mammalian fibrotic ECM response is less intense and restricted to the injury boundaries, where limited regeneration occurs for a limited amount of time after cardiac injury [17]. In the adult mammalian myocardium, inflammation, and metabolic genes have been identified as essential factors in cardiac regeneration [14]. In the following sections, we describe these differences and provide insights into underlying physiological, cellular, and molecular mechanisms.

### 2.1. Newts

Newts, in particular *Notophthalmus viridescens*, have the capacity to regenerate several of their tissues and organs, including the heart [35,36]. The newt heart consists of two atria and a single avascular ventricle, which can regenerate fully from a 20% resection of its apical region [14] or 10% resection of the total ventricular myocardium [13], without traces of scarring or impaired function. After resection, a blood clot is formed and regional myocardial contractions will close to minimize the leak and stabilize the seal (Figure 1) [13,14]. Within three days post-resection, cardiomyocytes in the damaged area show reduced expression of genes involved in contractile function, such as α-myosin heavy chain (αMHC) and cardiac troponins [14], indicating induction of dedifferentiation. By day seven, cardiomyocytes re-entered the cell cycle [14]. Furthermore, expression and secretion of fibrin and collagen genes, mainly in cardiac fibroblasts, are increased in the damaged area [13]. This results in impaired cardiac function and a 10% reduction in fractional shortening when compared to uninjured animals [13]. Within 21 days, the fibrin clot recedes, the epicardial layer is restored and cardiac function is improved [13]. Cellular proliferation is enhanced in the epicardium and cardiomyocytes of both atrial and ventricular walls [35]. The fibrin collagen matrix that covered the resected area is gradually repopulated with mononuclear infiltrates, followed by cardiomyocytes replacement at 60–90 days after the resection with transient scarring [13]. Interestingly, Gata4 has been detected in different cell populations during the regenerative process, indicating the reoccurrence of developmental cardiogenesis [13]. Laube et al. demonstrated that the expression of sarcomeric genes is down-regulated simultaneously in newt myocardium during this phase [37], suggesting that cardiomyocytes may likely undergo at least partial dedifferentiation in this stage.

### 2.2. Zebrafish

The zebrafish *Danio rerio* heart only consists of a single atrium and a single ventricle [22]. The ventricle is composed of three layers, including the epicardium, myocardium and endocardium [23], and is vascularized [22], thereby resembling the mammalian heart more than the newt heart. Zebrafish can regenerate their hearts after various injuries, such as surgical resection of the apex, cryoinjury, cardiomyocyte ablation, or hypoxia-reoxygenation damage [34,38,39,40]. The time needed for full cardiac regeneration depends on the type of injury. It takes about two months for resection of 20% of the ventricle for the heart to regenerate itself [41]. Similar to newts, resection of the ventricle leads to the formation of a blood clot within several seconds [34], which prevents excessive bleeding (Figure 1). One to three hours later, induced by inflammatory signals [42], endocardial cells round up and detach from underlying myofibers [42], creating large intercellular spaces. The typically aligned actin and myosin filaments become misaligned, and the transverse and longitudinal sarcomeric structures become visible due to its disorganization [11]. This state of endocardial activation is characterized by an increased permeability for inflammatory cells [11].

In some cardiomyocytes present in the outermost layer of the ventricle, the cortical muscle layer, Gata-4 expression is induced [15], which later localizes to the site of injury [15]. During regeneration of the damaged myocardium, epicardial cells contribute to blood vessel formation [43,44] and the endocardium drives cardiomyocyte proliferation by synthesizing retinoic acid (RA) [42]. Electrical conduction between the newly formed and preexisting cardiomyocytes develops within two to four weeks after resection [15]. After 30 days, the heart is almost fully regenerated, with most of the lost tissue replaced by vascularized and electrically coupled new cardiomyocytes [15] and only a small scar [11]. Lineage tracing experiments confirmed that newly formed cardiomyocytes indeed originate from preexisting, fully differentiated cardiomyocytes, rather than cardiac stem or progenitor cells [11]. Cardiomyocytes start proliferating post-injury, and the DNA replication was verified by 5-bromo-2-deoxyuridine (BrdU) labeling [34]. Zebrafish continue to grow until full sexual maturation, after which they still have a rather slow rate of cell turnover to their end of life [45]. This continuing cell turnover might be one of the reasons that the regenerative capacity remains during the lifespan of the zebrafish, unlike that in mammals wherein growth almost terminates at adulthood.

### 2.3. Mice

Mouse hearts show fibrosis in response to cardiac injury, Porrello et al. showed that the hearts of one-day old neonatal mice could still regenerate following local surgical resection [18]. Upon apical resection, the neonatal mouse heart, similar to zebrafish and newt hearts, shows the formation of a blood clot at the wound region, together with a robust inflammatory response. The clot is gradually absorbed and replaced with new cardiomyocytes. Within 21 days, a fully restored myocardium was observed. Two months later, the systolic function of the regenerated ventricular apex was almost completely restored. However, the cardiac proliferative capacity is lost in seven-day-old neonatal mice, who develop severe scarring of the infarcted region, leading to permanent myocardial damage, which is similar to the adult heart [18] (Figure 1). Genetic fate-mapping analysis in zebrafish studies implied that neonatal mouse cardiac regeneration is also a consequence of cardiomyocyte cell division [11]. Interestingly, it has been reported that cardiomyocytes in zebrafish and neonatal mouse hearts are mainly mononucleated with diploid nuclei (Figure 2). Whereas, in adult mouse and human hearts, the cells were shown to be binucleated with diploid nuclei or mononucleated with polyploid nuclei [15,46]. This phenomenon is widely accepted as a characteristic of terminal differentiation, leading to a permanent loss of proliferative capacity of postnatal cardiomyocytes.

### 2.4. Humans

Similar to observations in mice, neonatal human hearts appear to be capable of repairing myocardial injury and recovering cardiac function after myocardial infarction [16]. However, in adult human hearts a cardiac injury leads to pathological fibrosis rather than a regenerative response (Figure 1). Bergmann et al. described the first evidence for cardiomyocyte renewal in the adult human heart. They showed an annual cardiomyocyte turnover of about 0.8% at the age of 20. This number declines to 0.3% at the age of 75, while other cell types in the heart, especially endothelial and mesenchymal cells, show annual turnover rates of about 20% [10,26]. This suggests that the remaining proliferative capacity of human cardiomyocytes is minimal and not sufficient to replace lost cardiomyocytes and restore heart function after myocardial injury.

## 3. Four Fundamental Processes During Cardiac Regeneration

**Figure 3:**
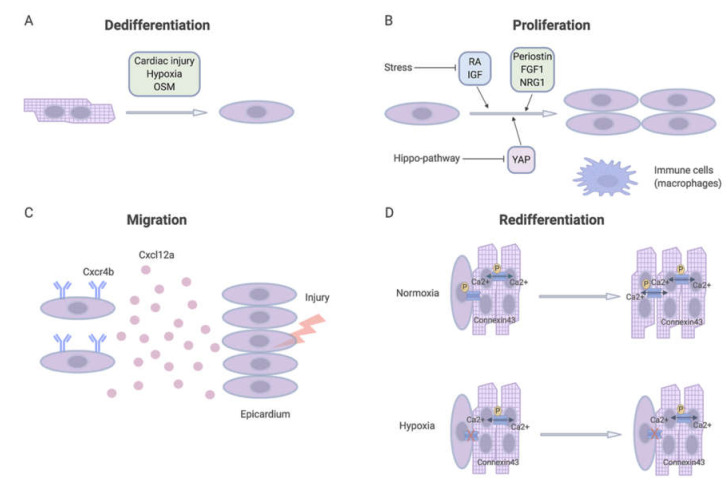
Four processes model of cardiac regeneration. **A**. Dedifferentiation. cardiac regeneration begins with dedifferentiation of preexisting differentiated cardiomyocytes after injury. It can be induced by hypoxia as well as OSM. **B**. Proliferation. After dedifferentiation, cardiomyocyte can start proliferation, which can be greatly enhanced by growth factors and cytokines as well as Hippo-YAP signaling pathway. **C**. Migration. Cardiac injury induce chemokine Cxcl12a expression and cardiomyocytes in the epicardium start to express its receptor Cxcr4b. Damage area enriched Cxcl12a expression stimulates the migration of cardiomyocytes toward the damaged area. **D**. Redifferentiation. When the newly formed cardiomyocytes arrive at the damaged area, they need to re-differentiate and become electrically coupled with the preexisting cardiomyocytes. OSM: Oncostain M; RA: Retinoic acid; IGF: Insulin-like growth factor; FGF1: fibroblast growth factor one; NRG1: Neuregulin 1.

In theory, cardiomyocytes that repopulate the injured area could stem from preexisting cardiomyocytes or from a progenitor/stem cell pool. Genetic fate-mapping experiments in zebrafish indicated that the regenerative ability of the heart mainly depended on the proliferation of preexisting cardiomyocytes. They displayed signs of dedifferentiation that exerted a vital role in the regenerative response [11,15]. Dedifferentiation is a cellular phenomenon in which highly specific cellular characteristics are lost, which consequently results in a relatively immature cellular state. For instance, dedifferentiated cardiomyocytes display disorganized sarcomeric structures, decreased contractile capacity, and re-expression of embryonic genes [42,44,47]. Upon ischemic myocardial injury, cardiomyocyte proliferation colocalized with regenerative molecules, such as RA and Insulin-like growth factor (IGF) [42,48]. In addition to local cardiomyocyte proliferation, cardiomyocytes migrate to the injured region. This migration may be regulated by, i.e., chemokine ligand Cxcl12 receptor Cxcr4 system, and once at the injury site, they re-differentiate to replace the lost or damaged tissue [15]. Thereby, it seems that cardiac regeneration is mediated by cardiomyocyte dedifferentiation, proliferation, migration and re-differentiation (Figure 3).

As briefly touched upon, many factors play a role in these four different phases of cardiac regeneration, including growth factors and ECM molecules. To get a better understanding of the various steps, individual factors will be reviewed in the following section.

### 3.1. Dedifferentiation Factors

#### 3.1.1. Hypoxia

Environmental oxygen concentration closely relates to zebrafish heart regeneration [49]. Chronic severe hypoxia in mice causes hypoxia-inducible factor-1α (HIF-1α) to be stabilized, which was sufficient to re-introduce the adult cardiomyocytes to the cell cycle and to increase the number of mononucleated cardiomyocytes. Three-month-old mice, subjected to LAD ligation and hypoxia for two weeks, exhibited significant improvement of systolic function and less fibrotic scar formation when compared to normoxia treated controls [50]. Conversely, hyperoxia or suppression of HIF-1α hinders heart regeneration following apical amputation in embryos and adult zebrafish [49].

A similar effect is seen with thyroid hormone inhibition. Both hypoxia and thyroid hormone inhibition induced downregulation of genes involved in fatty acid beta-oxidation, citric acid cycle (TCA), mitochondrial genes as well as muscle contraction genes, and upregulation of cell cycle genes. It is thought that this facilitates the generation of mononucleated cardiomyocytes [31,50].

#### 3.1.2. Oncostatin M

Oncostatin M (OSM) is an inflammatory cytokine that induces cardiac hypertrophy and cardiomyocyte survival [51]. The levels of OSM in the myocardium are increased upon cardiac injury [51]. Its signaling modulates ECM degradation and induces vascular endothelial growth factor (VEGF) expression in cardiomyocytes. In vivo and in vitro, OSM can induce dedifferentiation of cardiomyocytes [51], which initially is cardioprotective. Daily administration of OSM upon LAD ligation in mice exhibited an improved survival rate and better cardiac function, while a knockout of the OSM receptor leads to decreased survival and myocardial function [51]. Long-term activation of the OSM receptor, however, resulted in heart failure and increased mortality due to prolonged cardiomyocyte dedifferentiation [51]. OSM induced dedifferentiation is reversible both in vivo and in vitro in response to IGF1, among others, which causes the cardiomyocytes to re-differentiate into fully functional cardiomyocytes [52]

### 3.2. Proliferation Factors

Cell division is mediated via cytokinesis, including both the G1/S phase with DNA synthesis and the G2/M phase. Each phase transition is controlled by different complexes of cyclins and cyclin-dependent kinases [53,54]. The expression of these regulators is reduced in postnatal mammalian hearts, which is consistent with their exit from the cell cycle [28,55]. Heart development and chamber formation are mainly organized through cardiomyocyte division. However, this pattern of cardiomyogenesis plays an exceedingly limited role in adult mammals [28]. Since adult mammalian cardiomyocytes rarely re-enter the cell cycle, manipulations of cell cycle regulator genes, such as cyclins, kinases and cyclin-dependent kinases (CDK), have been performed [56,57,58] and some of the most effective approaches are highlighted in the following paragraph [59].

#### 3.2.1. Cyclins

Two cyclins that regulate the progression from G1- to S-phase are cyclin A2 and cyclin D [58,60,61,62]. Cyclin A2 is the only one not expressed in the postnatal mammalian heart, which makes it an exciting target. Induced cardiac expression of cyclin A2 in mice increased cardiomyocyte mitosis and cardiac hyperplasia [60], together with enhanced cardiac output [63]. Intramyocardial delivery of cyclin A2 in rats immediately after LAD ligation showed cardiomyocyte cell cycle activation in the infarct border zone [63]. Protein levels of Cyclin D1 and D3 decrease with age [64], which corresponds to the fact that adult mammalian cardiomyocytes do not re-enter the cell cycle [26]. When overexpressing Cyclin D1, mouse cardiomyocytes participate in the cell cycle, resulting in abnormal multinucleation [25,64]. After a permanent coronary artery occlusion increased cardiomyocyte proliferation and decreased infarct size was observed in cyclin D2 expressing mice [56,65]. Taken together, induced expression of cyclin A2 and cyclin D2 showed increased cardiomyocyte mitosis and improved heart function [60,63] and a decrease in infarct size [60], respectively, and thereby makes cyclins A and D promising targets for induction of cardiac regeneration.

#### 3.2.2. The Hippo-YAP Pathway

**Figure 4:**
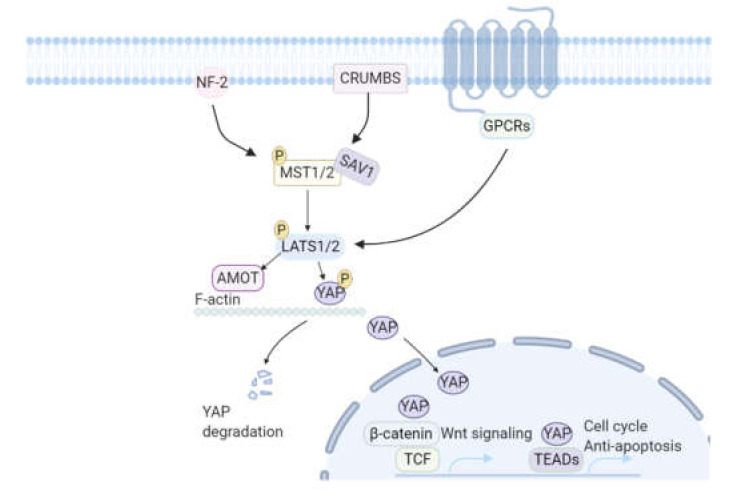
A simplified overview of the classic Hippo-YAP signaling pathway. The core of this Hippo-YAP is a cascade of kinase activation. When the Hippo pathway is activated by cell-cell contact, cytokine or mechanical stress, LATS1/2 and MST1/2 are recruited to the plasma membrane by NF-2 and scaffold protein Salvador (Sav) respectively. MST then activates LATS by phosphorylation. Active LATS then phosphorylates YAP and its binding protein angiomotin protein (AMOT) to inhibit nuclear localization of YAP. Phosphorylated AMOT, bound to YAP, dissociates from F-actin and translocates to the cytoplasm, where the complex will be degraded via the ubiquitin–proteasome system. Upon inactivation, YAP is dephosphorylated and translocated to the nucleus, where it interacts with TEAD transcription factors to promote cell cycle and anti-apoptosis gene transcription. Nuclear YAP can also enhance Wnt/β-catenin signaling by binding to b-Catenin/TCF transcription activation complex. NF-2: Neurofibromin 2; YAP: yes-associated protein 1; LATS1/2: Large tumor suppressor kinase ½; TEAD: TEA domain family member. The picture is created with BioRender.com.

The Hippo-YAP pathway is a conserved tumor-suppressor pathway [66,67] regulating cell polarity, cell adhesion, and ECM matrix deposition and is the major size-limiting pathway during organ growth. In addition, Hippo signaling participates in organ regeneration and tissue repair, including cell proliferation and survival [68,69,70,71]. When activated, LATS1/2 and MST1/2, the human homologs of the Hippo protein, are recruited to the plasma membrane by NF-2 and scaffold protein Salvador (Sav), respectively (Figure 4). MST triggers LATS by phosphorylation and subsequently, active LATS can phosphorylate YAP and its binding protein angiomotin protein (AMOT). Phosphorylation of YAP and AMOT prevents the nuclear localization of YAP. Phosphorylated AMOT, bound to YAP, dissociates from F-actin and translocates to the cytoplasm, where the complex is degraded via the ubiquitin–proteasome system. Upon inactivation, YAP is dephosphorylated and translocates to the nucleus, where it activates gene transcription of cell cycle and anti-apoptosis genes, together with TEAD transcription factors (Figure 4) [71,72].

Induced inhibition of the Hippo pathway or YAP overexpression caused increased cell cycle activity of cultured neonatal rat ventricular myocytes (NRVMs) and mouse cardiomyocytes. YAP overexpression in mice stimulated cardiac overgrowth [71,73], suggesting that YAP either stimulates cardiomyocyte proliferation or delays cell cycle withdrawal. Consistent with that, YAP inactivation resulted in significantly decreased cardiomyocyte proliferation and lethal cardiac hypoplasia [71].

However, healthy postnatal cardiac development was not affected by YAP1 deficiency [71], probably because of the switch from hyperplastic to hypertrophic heart growth within the first week of life [71]. Simultaneously with this switch, YAP expression decreased in the mouse heart. Inhibition of the Hippo pathway in cultured NRVMs revealed a modest increase in cell cycle activity [71]. Null alleles for Sav and LATS in 3-month-old mice also showed an increase in cardiomyocyte proliferation and an increase in the total number of mononuclear cardiomyocytes [68]. Furthermore, MST1 overexpression in mice caused increased cardiomyocyte apoptosis and decreased heart function, leading to death on postpartum day 15 [74]. Overall, this indicates that YAP is associated with hyperplastic rather than hypertrophic growth. Recently, Monroe et al. showed that forced overexpression of a constitutively active form of YAP in the adult mouse heart resulted in severe heart failure within 3 days. Cardiomyocytes from these animals displayed a more fetal-like phenotype and were able to proliferate, which indicates that YAP can induce adult cardiomyocytes to undergo partial dedifferentiation [75].

Given the fact that YAP expression is diminished soon after birth and that inhibition of the Hippo pathway induces proliferation and possibly dedifferentiation of cardiomyocytes, manipulation of the Hippo pathway seems a reasonable strategy to improve the regenerative capacity of the mammalian heart [68].

#### 3.2.3. P38 MAP Kinase

P38 MAP kinase (p38) negatively regulates the expression of several genes associated with mitosis, such as cyclin A and B. Overexpression of p38 blocks fetal cardiomyocyte proliferation both in vitro and in vivo. A cardiac-specific knockout of p38 in neonatal mice increased cardiomyocyte mitosis and sarcomere disassembly and upregulation of cyclin A2 expression [76]. Although p38 inhibition increased cardiomyocyte proliferation, it was insufficient to restore heart function after injury [76,77]. Inhibition of p38 in combination with FGF1 stimulation, induce partial dedifferentiation and downregulation of pro-apoptotic genes, result in improved proliferation and angiogenesis. Eventually, it leads to enhanced cardiac function, as compared with FGF1 administration only [76,77]. Thus, p38 inhibition in sequential combination with FGF1 might be an exciting strategy to re-introduce adult cardiomyocytes to the cell cycle.

#### 3.2.4. Meis1

Myeloid ecotropic viral integration site 1 (Meis1) plays an essential role in normal heart development [78], whereas chromosomal deletions of Meis1 were found in patients with congenital heart defects [79]. A knockout of Meis1 in mice resulted in smaller cardiomyocytes, enhanced cardiomyocyte mitosis and an increased percentage of mono-nucleated cells at postpartum day 14. Conditional knockouts in postpartum day 28 and seven-month-old mice also showed increased cardiomyocyte mitosis, meaning that a deficiency of Meis1 is sufficient to re-introduce adult cardiomyocytes to the cell cycle [78]. In accordance with this, Meis1 overexpressing mice presented bigger and less mitotically active cardiomyocytes. While wild type neonatal mice can regenerate their hearts after a MI, Meis1 overexpressing neonatal mice showed an impaired regenerative capacity [78]. Thus, Meis1 seems to be a negative regulator of cardiomyocyte proliferation and cardiac regeneration. This might be because Meis1 can activate CDK inhibitors p15, p16, and p21, which results in cell cycle arrest of cardiomyocytes [78,80].

#### 3.2.5. Extracellular Matrix Composition

The ECM plays an essential role in both cardiac development and cardiac regeneration by providing structural support and by direct signaling. During cardiac development in zebrafish, ECM signaling is essential for migration, proliferation and differentiation of cardiomyocytes by exerting mechanical properties. When cultured on rigid surfaces, rat and mouse neonatal cardiomyocytes displayed nuclear division without cell division, resulting in binucleated cells [81]. Cardiomyocytes cultured on compliant elastic matrices, on the other hand, showed signs of dedifferentiating cardiomyocytes and cell cycle re-entry [81].

Direct biological effects of the ECM became apparent after the administration of decellularized zebrafish ECM to adult mice that underwent a MI. Upon administration, the proliferation of the adult mouse cardiomyocytes increased, enabling cardiac regeneration and full functional recovery [82]. Similar experiments were performed with decellularized ECM from mouse hearts from postpartum day one (P1) and postpartum day seven (P7). Administration of P1 mouse ECM fragments to P1 and P7 mouse cardiomyocytes showed an increase in cell-cycle activity of both types of cardiomyocytes. However, when ECM from P7 mice was administered neither the P1 nor P7 cardiomyocytes revealed an increase in cardiomyocyte cell cycle activity. This suggests that ECM composition influences the cell cycle activity and that a change in ECM composition probably coincides with the loss of proliferative and regenerative capacity. Administration of P1 ECM in combination with broad matrix metalloproteinase (MMP) inhibitor, seemed to counteract the earlier observed increases in cardiomyocyte proliferation, which indicates the importance of MMPs to cardiac regeneration in response to injury [83]. In addition, inhibition of these MMPs in newts can result in failed limb regeneration, with malformed limbs or limb stumps and distal scars [84], indicating the essential role of MMPs in both the regenerative response and scar formation. In zebrafish, the expression of ECM components and modifying proteases also increases following injury. When TGF-β signaling, a potent regulator of ECM production, is inhibited, cardiac regeneration is blocked completely [85].

Apart from the biological effects, ECM rigidity could also regulate cardiomyocyte proliferation, for example, by regulating the nuclear localization of YAP [86]. A recent study by Bassat et al. [83] proposed a model that links ECM component Agrin to the Hippo-YAP pathway. During neonatal heart development in mice, the dystrophin-glycoprotein complex (DGC) connects ECM components, including Agrin, with the cytoskeleton. Upon Agrin binding, the DGC becomes unstable, which leads to disassembly of myofibrils, which is indicative for cardiomyocyte dedifferentiation. Within the first week after birth, Agrin levels decrease, allowing cardiomyocytes to differentiate and mature. Afterwards, YAP binds to the DGC, which keeps YAP away from the nucleus and thereby inhibits its function. When Agrin is administered, it binds to the DGC, causing YAP to dissociate from it. Simultaneously, increased nuclear YAP is seen upon Agrin administration in cardiomyocytes of adult mouse hearts after a MI. Nuclear YAP then induces cell cycle re-entry of the cardiomyocytes [83].

Periostin is another example of an ECM component that plays a pivotal role in cardiac development and epicardial-to-mesenchymal transition. Moreover, it can affect collagen formation and recruit macrophages [87,88]. Periostin is expressed throughout the whole heart in P2 mice in response to cardiac injury and increases cell-cycle activity of cardiomyocytes [89]. A knockout of Periostin in mouse hearts resulted in impaired angiogenesis, regeneration and increased fibrotic tissue formation in comparison to controls. While Periostin is necessary for cardiac regeneration, its expression level is insufficient to induce cardiac regeneration on its own [20].

#### 3.2.6. Neuregulin 1

Neuregulin 1 (NRG1) signaling is crucial for heart development by regulating proliferation, differentiation, maturation, and morphology of cardiomyocytes [90]. NRG1 is a member of the epidermal growth factor family and becomes upregulated in perivascular cells in response to injury. It binds with high affinity to the ErbB4 receptor, which is expressed on mature myocardium, together with ErbB2. As a result, ErbB4 dimerizes with ErbB2 [2,91], activating extracellular signal-regulated protein kinase (ERK) and AKT pathways, which results in regulation of differentiation, proliferation and cell migration [92]. In zebrafish, increased expression of NRG1 correlates with an increase in the number of proliferating cardiomyocytes during cardiac regeneration [2]. When NRG1 overexpression is induced for 30 days in the absence of injury, the zebrafish ventricular wall thickens approximately five folds [2]. In adult mice, constitutively active ErbB2 was able to reactivate cardiomyocyte proliferation [93].

#### 3.2.7. Neural Factors

Neural factors are also involved in cardiac regeneration. Classical experiments in newts showed that limb, lens, retina, and tail regeneration failed when they were not connected to an adequate number of nerve fibers. These nerve fibers guide regeneration via secretion of several nerve-derived factors, such as nerve growth factor (NGF). Cardiac denervation in zebrafish blunt the regenerative response leading to reduced cardiomyocyte proliferation [94].

Nerve-derived factor NGF plays an essential role in the maintenance and survival of sensory and sympathetic neurons [95], angiogenesis and cardiomyocyte survival after an AMI. Congestive heart failure patients showed reduced NGF expression in the heart [96], which might be due to reduced innervation density of sympathetic neurons [95]. Reduced NGF levels were also seen in a zebrafish model of heart failure and, upon NGF administration, cardiomyocyte proliferation increased and cardiac regeneration restored [96]. Neonatal mice with inhibited nerve innervation also showed impaired cardiac regeneration, which could be rescued by the administration of NGF and NRG1 [94]. Cultured dorsal root ganglion sensory neurons of chicks showed that NGF increased the expression of NRG1. As discussed before, NRG1 can re-introduce adult mammalian cardiomyocytes to the cell cycle and therefore the positive effect of NGF could be derived from the upregulation of NRG1 [96].

Another nerve-derived factor is FGF. Its signaling has been linked to regulation of tissue homeostasis, wound healing and tissue regeneration. It controls cytoplasmic remodeling and cell cycle re-entry via the mitogen-activated protein kinase (MAPK)/ERK pathway [15]. Activation of this pathway is essential for cellular differentiation and transformation [97]. When neonatal cardiomyocytes were stimulated with FGF1, genes involved in fetal development, regeneration, and cell cycle control were upregulated, together with a downregulation of pro-apoptotic genes [76]. Moreover, FGF1 administration improved angiogenesis in the regenerating hearts [77]. Thus, FGF1 expression contributes to dedifferentiation, by upregulation of fetal genes, and proliferation of cardiomyocytes. Additionally, FGF1 plays a role in the inhibition of apoptosis and stimulates angiogenesis, giving further support to the regenerating tissue.

#### 3.2.8. Retinoic Acid

RA is a morphogenetic factor essential for zebrafish cardiac development and regeneration [42] and induces DNA synthesis in cultured newt cardiomyocytes [98]. RA regulated production of FGF ligands and IGF by the epicardium promotes myocardium growth [99,100], thereby contributing to the morphogenesis of the ventricle during maturation. After apical resection, the zebrafish endocardium starts expressing Raldh2. Initially, Raldh2 is expressed in the whole heart, but gradually localizes to the damaged area. Raldh2 initiates RA synthesis from vitamin A. RA then stimulates cardiomyocyte proliferation and when RA signaling is blocked, cardiac regeneration will be impaired [42].

#### 3.2.9. IGF

IGF plays an essential role in heart growth and cardiomyocyte proliferation. Blocking IGF signaling in zebrafish embryos decreased the number of cardiomyocytes and impaired heart development. In response to apical resection, IGF2β is upregulated in the wound and the neighboring endo- and epicardium. Peak expression levels were observed between three to ten days post-resection [101], simultaneously with cardiomyocyte proliferation, which starts around one to two weeks post resection [11]. As mentioned before, a subset of cardiomyocytes expressing Gata4 start covering the wound area and proliferate during zebrafish cardiac regeneration. Blocking IGF signaling reduced Gata4 expression in the wound area and impaired cardiomyocyte mediated cardiac regeneration in zebrafish [101].

Acute stress in zebrafish results in failed regeneration and disabled replacement of fibrotic tissue with new myocardium. This is probably caused by a downregulation of a modulator of IGF signaling. Thus, stress-induced inhibition of the IGF signaling pathway causes a decrease in cardiomyocyte proliferation and thus impaired cardiac regeneration [102].

### 3.3. Migration Factors

After cardiomyocyte dedifferentiation and proliferation, chemokine-mediated cardiomyocyte migration to the site of injury is an essential step in zebrafish cardiac regeneration [103]. Molecular studies indicated that the Cxcl12a-Cxcr4b axis is highly conserved among different species and plays a vital role in cellular migration [104,105]. When part of the ventricle is amputated, Cxcl12a and Cxcr4b, a chemokine ligand and its receptor, start expressing in the damaged tissue and the epicardial cardiomyocytes, respectively (Figure 3). Cardiomyocytes migrate toward the injured area where Cxcl12a is highly enriched [106,107]. Inhibition of Cxcr4b with pharmacological intervention or by genetic deletion led to impaired cardiac regeneration following myocardial resection [103]. Although the cardiomyocytes were still able to proliferate, they could not migrate to the site of injury. This indicates the importance of molecular guidance for cardiomyocyte migration during successful cardiac regeneration.

#### Inflammation

The inflammatory response is essential for cardiac regeneration [23,108], because it regulates the survival of the remaining cardiomyocytes, removal of dead cells and debris, fibrosis and revascularization [109,110]. Macrophage depletion in newts lead to failed limb regeneration and extensive fibrosis [23,108]. A myocardial injury triggers a rapid inflammatory response in zebrafish [23,108], with the highest immune cell infiltration around three days post-resection [111]. Interestingly, the number of infiltrated inflammatory cells correlate with the mitotic activity in cardiomyocytes [108]. Moreover, inhibition of an inflammatory response strongly impaired regeneration of the heart after cryoinjury with reduced cardiomyocyte mitotic activity. Thus, the appropriate inflammatory response is necessary for cardiac regeneration in zebrafish [108].

Macrophage recruitment is indispensable for neonatal mouse cardiac regeneration and angiogenesis [109]. Macrophage depletion in neonatal mice resulted in failed cardiac regeneration and fibrotic scar formation [109]. Macrophages can be grouped into functionally different M1 or M2 populations according to the expression of the markers [112]. To explore the role of macrophages during cardiac regeneration, macrophages from P1 or P14 mice have been assessed after MI. In P14 mice M2 macrophage-specific gene was shown to be significantly upregulated, while expression of two out of three M1 macrophage-specific genes were unchanged or downregulated. In P1 mice, both types of macrophages were found in the heart, without any distinct bias toward M1 or M2. This indicates that although both P1 and P14 mice exhibited a macrophage response following MI, the degree and cellular polarization towards the M1 or M2 phenotype is different. Although the underlying mechanism is not yet fully understood, altered M1 or M2 polarization is considered to facilitate cardiac regeneration [109]. In addition, it is suggested that embryonic-derived cardiac macrophages from damaged neonatal hearts can facilitate cardiomyocyte proliferation and neovascularization with less inflammation. In contrast, recruited macrophages from injured adult hearts promote a strong inflammatory response and lead to a reduced capacity of cardiomyocyte proliferation and neovascularization [110]. Neonatal mice show neovascularization to support tissue renewal and restoration of cardiac function, while this neovascularization response does not occur in adult hearts upon injury [110]. An inflammatory response and inflammatory cell infiltration, in particular macrophages, are thus important to help wound healing and stimulate the regenerative response. Nonetheless, it is not clear to what extent the findings observed in neonatal mice can be deduced to other mammals, particularly humans. Up to now, the profile and characteristics of macrophages in human hearts has still not been explored comprehensively. In addition, whether human neonatal macrophages are conducive to facilitate myocardial regeneration was not reported yet. Interestingly, beneficial cardiac cell transplantation effects were suggested to be mediated via the macrophage polarization switch [113], leading to altered cardiac fibroblast activity and enhanced mechanical properties.

### 3.4. Re-Differentiation Factors

After dedifferentiation, proliferation and migration, re-differentiation of the cardiomyocytes occurs, which ensures that newly formed cardiomyocytes become electrically and mechanically coupled with the preexisting cardiomyocytes [15,114], resulting in fully functional regenerated cardiac tissue [51]. Redifferentiation of mouse adult cardiomyocytes is dependent on Ca^2+^ signaling from contracting NRVMs in co-culture. The formation of gap junctions is essential for intercellular Ca^2+^ signaling. Hypoxia induces dephosphorylation of connexin 43, an essential gap junction protein, inhibiting Ca^2+^ signaling and thus impairing adult cardiomyocyte re-differentiation. In vivo administration of an ischemia resistant isoform of connexin 43 showed enhanced re-differentiation of the newly formed cardiomyocytes and improved cardiac function after an MI in adult mice [115].

## 4. Conclusions

Several molecular mechanisms are shared among species that possess the ability to regenerate lost myocardium. These mechanisms include immune response, the different ECM composition, secreted factors from nerves, and regulation of the cell cycle as shown in Figure 3. Besides these well-established and somewhat general cellular and molecular mechanisms of regeneration, researchers are looking for additional and specific factors that are involved in cardiac regeneration. Mammalian impaired cardiac regeneration has led researchers to form several mutually non-exclusive hypotheses of why we lost our ability to regenerate our hearts.

Firstly, during birth, upon transition from the intrauterine environment (insufficient oxygen) to the postnatal environment, it is suggested that mitochondrial-derived reactive oxygen species (ROS) induced DNA damage leads to cardiomyocyte cell cycle arrest [116]. A slow reduction of inhaled oxygen concentration resulted in a decrease in mitochondrial metabolism and ROS levels in adult cardiomyocytes, which lead to less DNA damage. This subsequently contributed to more cardiomyocyte mitosis in the (non-)injured myocardium, as well as functional restoration post-MI [50]. These findings indicate that oxygen-dependent mitochondrial metabolism may be involved in the cell cycle arrest of cardiomyocytes [50].

A second hypothesis for the loss of mammalian cardiac regenerative capacity is built on the dynamic cardiomyocyte nuclear polyploidy. Cardiomyocytes of newts and zebrafish stay mononucleated and proliferative during their whole lifespan (Figure 2) [25,117]. Recent nucleation analysis of zebrafish hearts showed that 99% of zebrafish cardiomyocytes are mononucleated [118], and that they are, on average, smaller and contain fewer myofibrils than mammalian cardiomyocytes. Human cardiomyocytes are almost all mononucleated at birth, with only 25% of the cardiomyocytes being binucleated [10,26]. These cardiomyocytes are still able to proliferate in the first months of human life. However, during childhood the total DNA content per cell increases, resulting in tetraploid cardiomyocytes. This happens simultaneously with cardiomyocyte hypertrophy [10,26]. Mouse cardiomyocytes are also mononucleated and proliferative at birth, but they stop proliferating and 95% of the cardiomyocytes become binucleated within the first week after birth [18,26]. To test if there is a causal relationship between the proliferative capacity of cardiomyocytes and their DNA content, González-Rosa et al. induced polyploidy in zebrafish cardiomyocytes. When more than 45% of the cardiomyocytes were polyploid, cardiac regeneration failed and a fibrotic scar was formed after apical resection [118].

A third hypothesis refers to the development of the endotherm system which is regulated by thyroid hormone signaling. Using a phylogenetic analysis across 41 different species, Hirose et al. revealed that the abundance of mononucleated cardiomyocytes is inversely correlated with metabolic rate, body temperature, and thyroid hormone levels [31]. The level of thyroid hormone rises 50-fold 7 days after birth when the number of polyploidy cells increases and regeneration capacity drops [31]. Inhibition of the thyroid hormone pathway increased mononucleated cardiomyocyte numbers as well as their proliferative capacity. Transgenic mice expressing a dominant-negative form of the Thyroid Hormone Receptor alpha (THRα), the main cardiac-expressed receptor, displayed larger hearts, more mononucleated and smaller cardiomyocytes, prolonged cell cycle activity. Moreover, these transgenic mice retained their regenerative capacity for longer than 1 week after birth upon myocardial infarction [31]. Hirose et al. hypothesized that mammals acquire an endothermy system by elevated thyroid hormone levels. Even though a causal relationship has been suggested, the molecular mechanism is yet to be elucidated. Interestingly, thyroid hormone upregulation was observed in Xenopus to drive metamorphosis, with loss of cardiac regenerative capacity, although more than 80% of the cardiomyocytes stayed mononucleated [31,119]. This discrepancy between mammals and amphibians suggests that cardiomyocyte polyploidy might not be the only determining factor for cardiomyocyte regeneration. Instead, it might be the result of nucleus separation failure or blocked cell kinesis failure as a consequence of thyroid hormone-induced cardiomyocyte sarcomere rigidity [31,119].

There many more theories trying to identify why humans lose their cardiac regenerative capacity, including the role of oxidative stress-induced DNA damage, ECM deposition, inflammatory responses, cardiomyocyte polyploidy, and thyroid hormonal affected heart regeneration. By carefully examining these theories, we found overlapping or even shared cellular or molecular signatures. Cardiomyocyte contractile and lipid metabolism genes are always downregulated, while cell cycle genes are frequently upregulated as well as primitive gene signatures. Each theory might explain certain phases of the regenerative response and these phases together form the interlocked chain of the cardiac regeneration process.

For successful cardiac regeneration, cardiomyocytes need to go through the interlocked chain process of dedifferentiation, proliferation, migration, and re-differentiation and integration into the rest of the myocardium. Currently, most research is trying to study adult mammalian heart regeneration in only one part of the regenerative process (Figure 3). Little research has combined the introduction of multiple factors to induce cardiac regeneration. Since many factors are different in zebrafish, newt and neonatal mice than in adult mammalian hearts, the chance that only one factor can restore the regenerative capacity is small. Therefore, more research is needed to combine multiple factors, for example, using a combination of cardiac-specific activation of OSM to induce cardiomyocyte dedifferentiation, cardiac-specific inhibition of thyroid hormone signaling together with NRG1 and YAP activation to stimulate cardiomyocyte proliferation.

Most importantly, cardiomyocytes have to undergo dedifferentiation before proliferation can occur, which causes them to lose part of their contractile capacity. If this happens throughout the whole organ, cardiac function and integrity is jeopardized. Therefore, a more controlled and transient approach that targets a certain percentage of cardiomyocytes at specific locations might be necessary to progress modulation of cardiac regenerative capacity in humans towards therapeutic applicability.
